# Association between Diastolic Dysfunction with Inflammation and Oxidative Stress in Females ob/ob Mice

**DOI:** 10.3389/fphys.2017.00572

**Published:** 2017-08-23

**Authors:** Michelle Sartori, Filipe F. Conti, Danielle da Silva Dias, Fernando dos Santos, Jacqueline F. Machi, Zaira Palomino, Dulce E. Casarini, Bruno Rodrigues, Kátia De Angelis, Maria-Claudia Irigoyen

**Affiliations:** ^1^Hypertension Unit, Heart Institute (InCor), Faculdade de Medicina da Universidade de São Paulo São Paulo, Brazil; ^2^Translational Physiology Laboratory, Universidade Nove de Julho São Paulo, Brazil; ^3^Department of Integrative Immunological Cardiovascular Research, Institute for Neuro-Immune Medicine, Nova Southeastern University Fort Lauderdale, FL, United States; ^4^Nephrology Division, Department of Medicine, Universidade Federal de São Paulo São Paulo, Brazil; ^5^Department of Adapted Physical Activity, Faculty of Physical Education, Universidade Estadual de Campinas Campinas, Brazil

**Keywords:** obesity, cardiac dysfunction, autonomic, inflammation, female

## Abstract

**Objective:** To evaluate autonomic and cardiovascular function, as well as inflammatory and oxidative stress markers in ob/ob female mice.

**Methods:** Metabolic parameters, cardiac function, arterial pressure (AP), autonomic, hormonal, inflammatory, and oxidative stress markers were evaluated in 12-weeks female wild-type (WT group) and ob/ob mice (OB group).

**Results:** OB animals showed increased body weight, blood glucose, and triglyceride levels, along with glucose intolerance, when compared to WT animals. Ejection fraction (EF) and AP were similar between groups; however, the OB group presented diastolic dysfunction, as well as an impairment on myocardial performance index. Moreover, the OB group exhibited important autonomic dysfunction and baroreflex sensitivity impairment, when compared to WT group. OB group showed increased Angiotensin II levels in heart and renal tissues; decreased adiponectin and increased inflammatory markers in adipose tissue and spleen. Additionally, OB mice presented a higher damage to proteins and lipoperoxidation and lower activity of antioxidant enzymes in kidney and heart. Correlations were found between autonomic dysfunction with angiotensin II and inflammatory mediators, as well as between inflammation and oxidative stress.

**Conclusions:** Our results showed that female adult ob/ob mice presented discrete diastolic dysfunction accompanied by autonomic disorder, which is associated with inflammation and oxidative stress in these animals.

## Introduction

It is now well-established that both type 2 diabetes mellitus (DM) and obesity result from a combination of genetic factors and lifestyle (James, 1995; Must et al., 1999). It should be noted that the incidence of cardiovascular disease (CVD) in diabetic individuals with one risk factor is equivalent to the risk of a non-diabetic individual with the association of three risk factors (Abraham, 2003).

Similarly to observed in men, CVD is the major cause of mortality in women, especially after menopause (Go et al., 2014). It is important to note that DM substantially increases mortality risk (Kannel et al., 1976; Kaseta et al., 1999). Recently, an elegant study from Italy, showed that diabetes increased the risk of first ever ischemic stroke by more than 50% in both men and women (Policardo et al., 2015). Interestingly, postmenopausal women at the age of 55–74 years presented a higher risk for ischemic stroke than men of comparable age. Indeed, females have a higher risk of developing cardiovascular complication due to diabetes than male. Impairment on endothelial response due to diabetes is more evident in women than in males, which alters the positive hemodynamic estrogen effects by complex interactions between insulin and estrogen signaling. Hyperglycemia unfavorably alters the balance of expression and activity of estrogen receptors (Dantas et al., 2012). Furthermore, an increased oxidative stress and endothelin-1 can be observed, which contribute to promoting vasoconstriction and platelet aggregation (Cardillo et al., 2002) and decreases endothelium-dependent relaxation and NO production via impaired insulin signaling (Dantas et al., 2012). Additionally, diabetic females present higher susceptibility to diabetic cardiopathology as well as CHD. Kiencke et al. (2010) showed that preclinical diabetic cardiomyopathy was common in diabetic patients, with female sex being the only independent predictor of LV hypertrophy. However, our knowledge of CVD and its mechanisms is largely based on studies involving male individuals. Therefore, it is essential to establish strategies for the treatment of CVD in women in order to reduce the morbidity and mortality rates among them.

The association between bioactive molecules released from adipose tissue and oxidative stress may be responsible, at least in part, for insulin resistance and endothelial dysfunction (Janszky et al., 2004). In this sense, in recent years, the immune system (mainly related to increase of pro-inflammatory cytokine levels) has been the focus of several studies on the development and prognosis of coronary artery disease and CVD risk (Ridker et al., 2000a,b; Pradhan et al., 2002; Janszky et al., 2004). However, the mechanisms underlying the impact of female gender on autonomic dysfunction, inflammation and oxidative stress associated with end organ damage induced by obesity/diabetes are still unknown.

A model of type 2 diabetes that has been widely studied is the ob/ob mice (leptin deficiency), as their morbid obesity and type 2 diabetes are similar to those seen in humans (Coleman, 1978). The ob/ob mice present obesity, hyperglycemia, insulin resistance, diabetic neuropathy (Hasty et al., 2001; Drel et al., 2006), as well as decreased baroreflex sensitivity (Hilzendeger et al., 2010). Therefore, since the association between diabetes and obesity considerably increases the risk of CVD and most studies with CVD have been restricted to male subjects, more research involving female individuals should be carried out. Thus, the aim of this study was to evaluate cardiovascular function, autonomic modulation, inflammatory and oxidative stress markers in ob/ob female mice. We tested the hypothesis that ob/ob female mice present an impairment on cardiovascular and autonomic parameters, as well as an increased oxidative stress and inflammatory markers when compared to the wild type, and that this model of diabetes and obesity could be used for further studies regarding cardiovascular changes in this condition. Thus, this work is justified by the lack of exploratory mechanistic studies in female ob/ob mice.

## Materials and methods

Twelve-week-old female wild-type C57Bl6/J and leptin-deficient C57Bl6/J ob/ob mice were obtained from the Animals Facilities of the Federal University of Sao Paulo. The mice were divided into wild-type group (WT; *n* = 14) and ob/ob group (OB; *n* = 14). All surgical procedures and protocols were approved by the ethics committee of the Universidade Nove de Julho (AN0021/2014) and were conducted in accordance with the Guide for the Care and Use of Laboratory Animals, issued by the National Institutes of Health. All *in vivo* evaluations and euthanasia were conducted on non-ovulatory phase of estrous cycle, once it is well-documented in the literature that the different phases of estrous cycle, as well as the ovarian hormone deprivation may lead to significant adjustments on cardiovascular physiology in females (Saleh et al., 2000; Flues et al., 2010; Kuo et al., 2010). The tissues were harvested 24 h after the hemodynamic measurement.

### Metabolic evaluations

Blood glucose, triglyceride, and cholesterol concentrations were measured (Accucheck and Accutrend, Roche), after 4-h fasting. For glucose tolerance test (GTT), mice were fasted, with animals receiving only water, for 6 h. Blood samples were taken from a tail cut at 0, 15, 30, 60, and 90 min after i.p. glucose load (1.5 g/kg). Blood glucose was determined by Accu-Chek Advantage Blood Glucose Monitor (Roche Diagnostic Corporation, Indianapolis, IN).

### Echocardiographic evaluation

Echocardiography was performed by an observer blinded to the groups, according to the Guidelines of the American Society of Echocardiography. Mice were anesthetized (ketamine–xylazine 80:40 mg/kg i.p.), and images were obtained using a Sequoia 512 ultrasound system (ACUSON, Mountain View, CA, USA) with a 10–14 MHz linear transducer for the measurement systolic function [ejection fraction (EF) and fractional shortening (FS)] and diastolic function [left ventricular isovolumetric relaxation time (IVRT) and E wave/A wave ratio (E/A)] and global cardiac function [myocardial performance index (MPI)] (Wichi et al., 2007).

### Hemodynamic measurements

One day after echocardiographic evaluation, mice were anesthetized (ketamine–xylazine 80:40 mg/kg i.p.) and polyethylene-tipped Tygon cannulas (4 cm of PE-08 connected to 2 cm of PE-50, Clay Adams) filled with heparinized saline were inserted into the carotid artery and jugular vein for direct measurements of arterial pressure (AP) and drug administration, respectively. Twenty-four hours after the cannulation procedure, the arterial cannula was connected to a transducer (Blood Pressure XDCR, Kent^©^ Scientific), and blood pressure signals were recorded for a 20-min period using a microcomputer equipped with an analog-to-digital converter (CODAS, 4-kHz sampling frequency, Dataq Instruments), being the animals conscious, under no anesthesia effect. The recorded data were analyzed on a beat-to-beat basis to quantify changes in AP and heart rate (HR) (Heeren et al., 2009).

Baroreflex sensitivity was evaluated by a mean index relating the tachycardic or the bradycardic responses to mean AP changes (~30–40 mmHg), induced by increasing doses of sodium nitroprusside (100–250 ng/kg body weight i.v.) and phenylephrine (80–250 ng/kg body weight i.v.) injections, respectively, being the animals conscious, under no anesthesia effect, right after the hemodynamic recording (Heeren et al., 2009).

### Autonomic modulation

#### Linear analyses

Time-domain analysis consisted in calculating mean pulse interval (PI) variance from its respective time series. For frequency domain analysis, the whole 20-min time series of PI and systolic arterial pressure (SAP) were cubic-spline-interpolated and decimated to be equally spaced in time. Following linear trend removal, power spectral density was obtained by the Fast Fourier Transformation. Spectral power for low- (LF: 0.1–1.0 Hz), and high- (HF: 1.0–5.0 Hz) frequency bands was calculated by means of power spectrum density integration within each frequency bandwidth, using a customized routine (MATLAB 6.0, Mathworks) (Heeren et al., 2009).

#### Non-linear analyses

##### Poincaré plot analysis

The Poincaré plot is a graph in which each R-R interval is plotted as a function of the previous R-R interval. Briefly, scattergrams of successive R-R intervals were plotted for the entire period, and the SD of instantaneous R-R interval variability and the SD of continuous variability (SD2) were then analyzed. The SD1 is an index of the instantaneous recording of the variability of beat-to-beat and represents the parasympathetic activity, whereas the SD2 index represents the long-term HRV and reflects the overall variability (Raimundo et al., 2013).

#### Detrended fluctuation analysis

The detrended fluctuation analysis technique was used to quantify the fractal scaling properties of short- and intermediate-term R-R interval time series. The root-mean-square fluctuation of integrated and detrended time series is measured at different observation windows and plotted against the size of the observation window on a log-log scale. The details of this method have been described elsewhere (Lombardi et al., 1996).

#### Approximate entropy and sample entropy

Approximate entropy (ApEn) was proposed by Pincus in 1991 as a method for measuring regularity and complexity in time series, and has been successfully used to analyze physiological time series (Pincus, 1991). The sample entropy (SampEn) was used to assess the complexity of the HR “signal” under the different conditions. SampEn measures the likelihood that runs of patterns that are close to each other will remain close in the next incremental comparisons (Pincus, 1991).

### Angiotensin II and angiotensin 1–7 evaluations

Angiotensin levels were analyzed by a high-performance liquid chromatography (HPLC). The heart, kidney, and adipose tissues were homogenized with 100 mM sodium phosphate buffer, 340 mm sucrose, and 300 mM NaCl at pH 7.2. The samples were concentrated on C_18_Sep-Pak column, activated with methanol (5 mL), tetrahydrofuran (5 ml), hexane (5 mL), methanol (5 mL), and water (10 mL), as previously described (Ronchi et al., 2007).

### Determination of inflammatory mediators

Interleukin-1 (IL-6), plasminogen activator inhibitor-1 (PAI-1), tumor necrosis factor-α (TNF-α), and adiponectin levels in the adipose tissue and spleen were determined using a commercially available ELISA kit (R&D Systems Inc.), in accordance with the manufacturer's instructions. ELISA was performed in 96-well polystyrene microplates with a specific monoclonal antibody coating. Absorbance was measured at 540 nm in a microplate reader (da Palma et al., 2016).

### Oxidative stress profile assessment

The heart (ventricles) and kidney were cut into small pieces, placed in ice-cold buffer, and homogenized in an ultra-Turrax blender with 1 g tissue per 5 mL 150 mM KCl and 20 nM sodium phosphate buffer, pH 7.4. The homogenate was centrifuged at 600 g for 10 min at −26°C. All procedures were described in detail elsewhere (da Palma et al., 2016).

Briefly, analysis followed the protocols:

*Lipoperoxidation evaluated by thiobarbituric acid reactive substances (TBARS):* For the TBARS assay, trichloroacetic acid (10%, w/v) was added to the homogenate to precipitate proteins and to acidify the samples. This mixture was then centrifuged (10,000 g, 3 min), the protein-free sample was extracted, and thiobarbituric acid (0.67%, w/v) was added to the reaction medium. The tubes were placed in a water bath (100°C) for 15 min. The absorbencies were measured at 535 nm using a spectrophotometer.
*Determination of protein oxidation using the protein carbonyl assay:* Tissue samples were incubated with 2,4-dinitrophenylhydrazine (DNPH 10 mM) in a 2.5 M HCl solution for 1 h at room temperature in the dark. Samples were vortexed every 15 min. Subsequently, a 20% trichloroacetic acid (w/v) solution was added and the solution was incubated on ice for 10 min and centrifuged for 5 min at 1,000 g to collect protein precipitates. An additional wash was performed with 10% trichloroacetic acid (w/v). The pellet was washed three times with ethanolethyl acetate (1:1) (v/v). The final precipitates were dissolved in 6 M guanidine hydrochloride solution and incubated for 10 min at 37°C, and the absorbance was measured at 360 nm.
*Antioxidant enzyme activities:* The quantification of SOD activity, expressed as U/mg protein, was based on the inhibition of the reaction between O_2_^·−^ and pyrogallol. CAT activity was determined by measuring the decrease inH_2_O_2_ absorbance at 240 nm.

### Statistical analysis

Data are expressed as mean ± SEM. The normality of data was tested by Kolmogorov–Smirnov. Student *t*-test was used to compare groups. Pearson correlation was used to analyze the association between variables. For the correlation analysis, it was used between 5 and 7 animals per group. The significance level was established at *P* ≤ 0.05.

## Results

### Metabolic and echocardiographic evaluations

At the end of the protocol, body weight, abdominal adipose tissue, glycemia, triglycerides, and GTT were higher in the OB group than in the WT group. No differences were observed regarding total cholesterol between the groups (Table 1).

**Table 1:** Body weight, tissues weight, and metabolic evaluations in wild type group (WT; *n* = 14) and ob/ob group (OB; *n* = 14).

	**WT**	**OB**
Body weight (g)	20.1 ± 0.2	50.2 ± 0.5^*^
Abdominal adipose tissue (g)	0.41 ± 0.01	8.02 ± 0.20^*^
Glycaemia (mmol/L)	6.71 ± 0.33	9.99 ± 0.55^*^
Tryglicerydes (mg/dL)	107 ± 13	142 ± 6^*^
Cholesterol (mg/dL)	162 ± 2	167 ± 3
GTT (mg/dL/min)	15, 424 ± 3, 351	38, 491 ± 1, 272^*^

Data are reported as mean± SEM.

* *p < 0.05 vs. WT*.

**Figure 1:**
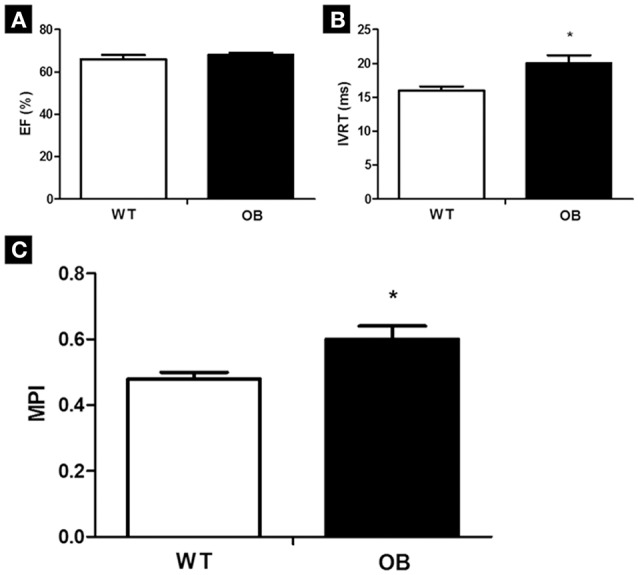
Cardiac function in WT (*n* = 14) and OB (*n* = 14). **(A)** Ejection fraction (EF); **(B)** isovolumetric relaxation time (IVRT); **(C)** myocardium performance index (MPI) groups. ^*^p < 0.05 vs. WT.

Echocardiographic examinations showed that WT and OB groups presented similar systolic function, since that no difference was observed in EF (Figure 1A) and FS (WT: 30 ± 1 vs. OB: 32 ± 1%) parameters. However, the OB mice presented increased values of IVRT when compared to WT mice (Figure 1B), despite the values of E/A ratio were similar between the groups (WT: 1.81 ± 0.13 vs. OB: 1.44 ± 0.06). The global cardiac function, evaluated by the myocardial performance index (an index of cardiac stress), demonstrated increased values in OB group when compared to WT group (Figure 1C).

### Hemodynamic and autonomic assessments

**Figure 2:**
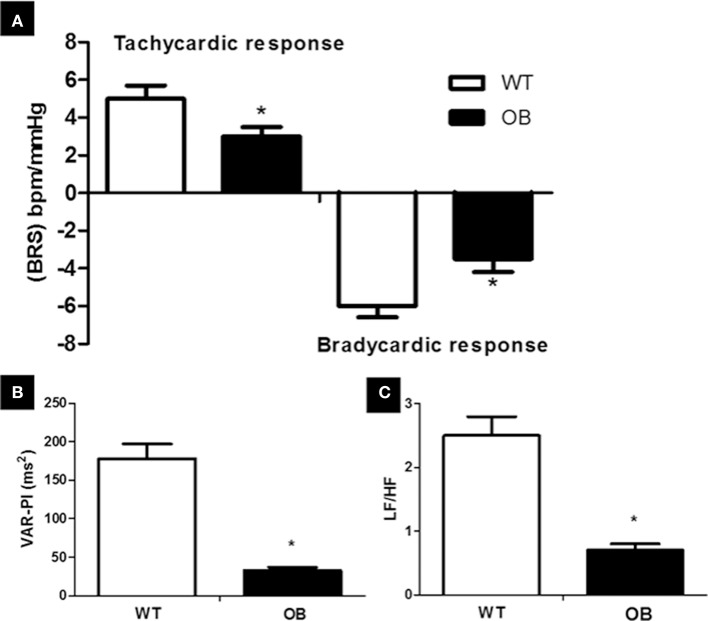
Cardiovascular autonomic assessment in WT (*n* = 8) and OB (*n* = 8) groups. **(A)** baroreflex sensitivity evaluated by bradycardic and tachycardic responses to arterial pressure changes; **(B)** total variance of the pulse interval; **(C)** sympathovagal balance. ^*^p < 0.05 vs. WT.

There were no differences in the direct hemodynamic measurements between the groups regarding the systolic, diastolic, and mean APs as well as HR (Table 2). Baroreflex sensitivity, evaluated for both tachycardic responses and bradycardic responses was impaired in the OB group when compared to the WT group (Figure 2A).

**Table 2:** Hemodynamic and autonomic parameters in wild type group (WT; *n* = 8) and ob/ob group (OB; *n* = 8).

	**WT**	**OB**
**HEMODYNAMIC PARAMETERS**		
SAP (mmHg)	132 ± 2	129 ± 4
DAP (mmHg)	96 ± 2	97 ± 3
MAP (mmHg)	114 ± 2	114 ± 4
HR (bpm)	562 ± 14	564 ± 20
**HEART RATE VARIABILITY**		
RMSSD (ms)	12.0 ± 1.3	6.1 ± 0.9^*^
LF (ms^2^)	135 ± 26	7 ± 2^*^
HF (ms^2^)	43 ± 6	13 ± 2^*^
**ARTERIAL PRESSURE VARIABILITY**		
VAR (mmHg^2^)	20 ± 3	24 ± 3
LF (mmHg^2^)	9 ± 1	10 ± 1
**BAROREFLEX SENSITIVITY**		
Alpha index (ms/mmHg)	3.12 ± 0.32	0.74 ± 0.07^*^
**NON-LINEAR ANALYSES**		
SD1	13.9 ± 2.5	6.0 ± 1.3^*^
SD2	24.2 ± 1.5	15.0 ± 2.3^*^
Alpha 1	1.00 ± 0.09	0.57 ± 0.05^*^
Alpha 2	0.65 ± 0.04	0.95 ± 0.03^*^
SampEn	1.56 ± 0.09	1.72 ± 0.03
ApEn	1.41 ± 0.05	1.47 ± 0.02

Data are reported as mean± SEM.

* *p < 0.05 vs. WT. SAP, systolic arterial pressure; DAP, diastolic arterial pressure; MAP, mean arterial pressure; HR, heart rate; RMSSD, root mean square of the successive differences; VAR, total variance; LF, low-frequency band; (0.1–1.0 Hz); HF, high-frequency band (HF: 1.0–5 Hz); Poincare plot (SD1 and SD2); Detrended fluctuation analysis (Alpha 1 and Alpha 2); Sample entropy (SampEn), and Approximate entropy (ApEn)*.

Regarding HR variability, there was a decrease in RMSSD index, LF and HF bands in OB group when compared to the WT group (Table 2). The OB group exhibited a reduction of PI variance (VAR-IP) and LF/HF ratio when compared to the WT group (Figures 2B,C).

There were no differences between the groups regarding variability in systolic blood pressure (SAP-VAR). Additionally, the LF band of systolic blood pressure (SAP-LF) was similar in both WT and OB groups (Table 2). Moreover, spontaneous baroreflex sensitivity, evaluated by alpha index, was reduced in OB group when compared to WT group (Table 2), confirming the results obtained from tachycardic and bradycardic responses.

When evaluated autonomic modulation by non-linear analyses, SD1, SD2, and Alpha 1 parameters were reduced, while Alpha 2 was increased in OB animals as compared with WT (Table 2).

### Determination of angiotensin II and 1–7 levels

**Figure 3:**
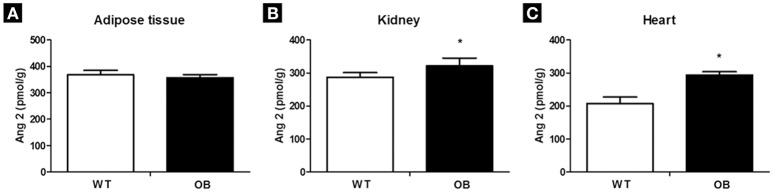
Angiotensin II levels in WT (*n* = 6) and OB (*n* = 6) groups. **(A)** adipose tissue; **(B)** kidney; **(C)** heart. ^*^p < 0.05 vs. WT.

There were no differences regarding the levels of angiotensin II (Figure 3A), and angiotensin 1–7 between the WT and OB groups in the adipose tissue. Regarding angiotensin dosed in the kidney, a decrease in angiotensin 1–7 in the OB group was observed when compared to the WT group. In addition, there was an increase in angiotensin II in the OB group when compared to WT group (Figure 3B).

In the left ventricle, the OB group showed an increase in angiotensin 1–7 when compared to the WT group. Moreover, the OB group presented an increase in angiotensin II when compared to the WT group (Figure 3C).

### Evaluation of inflammatory and hormonal markers

**Figure 4:**
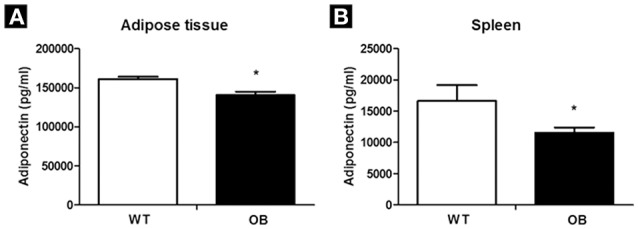
Adiponectin levels in WT (*n* = 6) and OB (*n* = 6) groups. **(A)** Adipose tissue; **(B)** spleen. ^*^p < 0.05 vs. WT.

The OB group showed lower adiponectin values when compared to the WT group in the adipose tissue (Figure 4A). Regarding IL-6, no differences in adipose tissue were found between the studied groups. We observed an increase in PAI-1 in the OB group when compared to the WT group (Table 3).

**Table 3:** Inflammatory markers in abdominal adipose tissue and spleen in wild type group (WT; *n* = 6) and ob/ob group (OB; *n* = 6).

	**WT**	**OB**
**ABDOMINAL ADIPOSE TISSUE**		
IL-6 (pg/ml)	154 ± 7	141 ± 16
PAI-1 (pg/ml)	1, 353 ± 211	4, 616 ± 710^*^
**SPLEEN**		
TNF alpha (pg/ml)	70 ± 5	96 ± 4^*^
IL-6 (pg/ml)	148 ± 15	380 ± 52^*^
PAI-1 (pg/ml)	1, 577 ± 407	3, 706 ± 723^*^

Data are reported as mean ± SEM.

* *p < 0.05 vs. WT*.

Adiponectin in the spleen was lower in the OB group when compared to the WT group (Figure 4B). We observed an increase in TNF-alpha in the OB group spleen tissue when compared to the WT group. Furthermore, the OB group had elevated levels of IL-6 in the spleen, as well as an increased PAI-1 levels in the OB group when compared to the WT group (Table 3).

### Oxidative stress investigation

Regarding renal oxidative stress, protein oxidation (carbonyls) was increased in the OB group when compared to the WT group. Additionally, lipoperoxidation (TBARS) was higher in the OB group when compared to the WT group. There was no difference in SOD between the WT and OB groups. The OB group showed a reduction in CAT when compared to the WT group (Table 4).

**Table 4:** Renal and cardiac oxidative stress in wild type group (WT; *n* = 8) and ob/ob group (OB; *n* = 8).

	**WT**	**OB**
**RENAL**		
Protein oxidation (nmol/mg protein)	4.8 ± 0.7	6.4 ± 0.5^*^
Lipoperoxidation (μmoles/mg protein)	8.8 ± 1.2	11.0 ± 0.8^*^
SOD (USOD/mg protein)	19 ± 1	18 ± 1
CAT (nmol/mg protein)	3.5 ± 0.3	2.4 ± 0.2^*^
**CARDIAC**		
Protein oxidation (nmol/mg protein)	3.2 ± 0.2	4.3 ± 0.4^*^
Lipoperoxidation (μmoles/mg protein)	4.3 ± 0.6	5.7 ± 0.3^*^
SOD (USOD/mg protein)	10 ± 1	10 ± 1
CAT (nmol/mg protein)	0.3 ± 0.02	0.3 ± 0.03

Data are reported as mean± SEM.

* *p < 0.05 vs. WT*.

Cardiac protein oxidation and lipoperoxidation were increased in the OB group when compared to the WT group. There were no differences between groups in cardiac levels of the antioxidant enzymes SOD and CAT (Table 4).

### Correlation analysis

**Figure 5:**
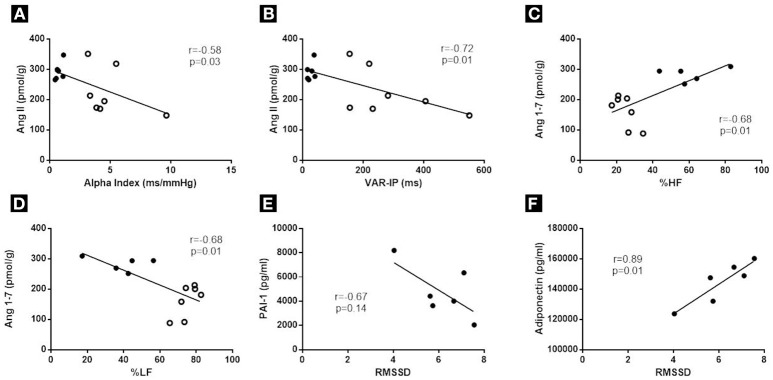
Correlations between: **(A)** Alpha Index and cardiac Ang II; **(B)** VAR-IP and cardiac Ang II; **(C)** %HF and cardiac Ang 1–7; **(D)** %LF and cardiac Ang 1–7; **(E)** RMSSD and PAI-1; **(F)** RMSSD and Adiponectin. It was used between 5 and 7 animals per group. The correlations E and F were made using only the OB group. Open circles represent the WT group and closed circles represent the OB group.

Positive correlations were found between baroreflex sensitivity and inflammatory mediators/or hormonal markers: tachycardic (*r* = −0.81) and bradycardic responses (*r* = 0.75) were correlated with IL-6 in spleen tissue; the alpha index was positively correlated with adiponectin in the spleen tissue (*r* = 0.57) and was inversely correlated with PAI-1 in the spleen tissue (*r* = 0.65) and with angiotensin 2 in the left ventricle (*r* = −0.58) (Figure 5).

Moreover, indexes of cardiac autonomic modulation were associated with hormonal, inflammatory and oxidative stress markers: RMSSD was positively correlated with adiponectin (*r* = 0.86) and was negatively correlated with spleen levels of PAI-1 (*r* = −0.81) and cardiac levels of angiotensin II (*r* = −0.68); VAR PI was inversely correlated with cardiac levels of angiotensin II (*r* = −0.72); %LF was inversely correlated with cardiac levels of angiotensin 1–7 (*r* = −0.68); and %HF was positively correlated with cardiac levels of angiotensin 1–7 (*r* = 0.68) (Figure 5).

Spleen levels of TNF-alpha were negatively correlated with angiotensin 1–7 in cardiac (*r* = −0.75) and renal tissues (*r* = −0.76) and were positively correlated with angiotensin II levels in renal tissue (*r* = 0.92) and cardiac lipoperoxidation (*r* = 0.64). The spleen levels of PAI-1 were also directly correlated with cardiac protein oxidation (*r* = 0.60) and lipoperoxidation (*r* = 0.64) as well as with renal lipoperoxidation (*r* = 0.84).

Considering only the OB group, it was observed a positive correlation between RMSSD and adiponectin (*r* = 0.89) and a negative correlation between RMSSD and PAI-1 (*r* = −0.67) Figure 5.

## Discussion

Results of this study showed marked metabolic dysfunction in female obese diabetic mice. In addition, cardiac dysfunctions, impaired baroreflex sensitivity, and HRV, as well as increased inflammatory profile and oxidative stress markers were displayed by OB group as compared with WT group.

The ob/ob mice are characterized by hyperphagia caused by the absence of leptin (Coleman, 1978; Zhang et al., 1994) accompanied by an increase in a fat deposition (Coleman, 1978; Verploegen et al., 1997). In our research, a significant increase in body weight, blood glucose, and glucose intolerance. The increase in body weight, besides being associated with metabolic negative changes, directly affects cardiac structure, and function in women (Kannel et al., 1976; Kaseta et al., 1999). Concerning echocardiographic evaluations, although systolic function was similar between experimental groups, an increased IVRT and an impairment in myocardial performance index were observed in ob/ob female mice. Thus, it is possible that, at this age, the animals are beginning to develop left ventricle diastolic dysfunction, since that only IVRT was changed. Left ventricular diastolic dysfunction reflects an impairment of the filling properties of the left ventricle, being associated with the future development of heart failure in different populations (Bella et al., 2002). An increase of body mass index (Russo et al., 2011), as well as reduced glucose tolerance (Shimabukuro et al., 2011) were associated with worse left ventricle diastolic function, independent of ventricle mass and associated risk factors.

In fact, diabetes appears to attenuate the possible cardioprotective effects of ovarian hormones. Regarding the mechanisms associated with ventricular dysfunction, comparing males and females db/db mice, Bowden et al. (2015) revealed that diabetes-induced upregulation of both hypertrophic and pro-oxidant gene expression in females. Additionally, changes in insulin signaling, oxidative stress, mitochondrial dysfunction, inflammatory status, renin angiotensin system, cardiac glycogen metabolism (Reichelt et al., 2013), and myocardial calcium handling (Regitz-Zagrosek et al., 2010) can be pointed out as potential mechanisms associated with diabetic females cardiac dysfunction.

In order to expand our knowledge, we investigate if the diastolic dysfunction in ob/ob female mice was associated with cardiovascular autonomic modulation. Regarding this issue, the OB group demonstrated important signals of autonomic dysfunction, as observed by reduction of RMSSD, HF, and LF bands of PI variability. These autonomic changes were possibly associated with the impairment of baroreflex sensitivity observed in this group, being a permissive element to the establishment of primary changes in autonomic modulation in males rats (Irigoyen et al., 1995).

The imbalance between sympathetic and parasympathetic modulation may also be associated with renin-angiotensin system stimulation, leading to the AT1 receptor activation (Campagnaro et al., 2012). Indeed, in our study, although we did not find any significant AP differences between WT and OB groups, obese animals showed a decrease in baroreflex sensitivity accompanied by with an increase in angiotensin II levels in the heart and in the kidney. Thus, our findings suggest an increased activation of the constrictor axis of the system may be related to increased risk of disorders of autonomic modulation. In fact, our results demonstrated inverse correlations between RMSSD, VAR IP, and alpha index with angiotensin II levels in the cardiac tissue.

Moreover, it is plausible to consider that the hyperglycemia altering negatively the balance of expression and activity of estrogen receptors (Dantas et al., 2012), which hold a positive role against the deleterious effects of angiotensin II (Xue et al., 2007), may be contributing to the elevated levels of angiotensin II and reduced levels of angiotensin 1–7 in the kidney of OB group when compared to the WT group. Similar changes in angiotensin II and angiotensin 1–7 levels have been associated with the development of different diseases (Campagnaro et al., 2012), and contributing to the cardiovascular and autonomic dysfunction (Dzau and Re, 1994; Silva et al., 2015; Srinivasa et al., 2015). It is interesting to note that, in the left ventricle, OB mice presented an increase in angiotensin II, as well as in angiotensin 1–7 levels, suggesting a possible counter-regulatory mechanism in order to maintain cardiac homeostasis. The angiotensin II/ACE/AT1 receptor axis activation leads to vasoconstriction of arterioles, resulting in decreased blood flow, which may affect glomerular filtration. This impairment in glomerular filtration may increase the production of inflammatory cytokines and reactive oxygen species, leading to increased oxidative stress (Silva et al., 2015).

It is important to emphasize that some studies have shown that the vagal nerve can modulate the inflammatory and oxidative stress responses in some pathophysiological situations (Van Gaal et al., 2006). In the present study, we chose to assess the inflammatory status in spleen and adipose tissue, since that are important regulators of neuro-immune-endocrine function (Van Gaal et al., 2006; Lau, 2010). A decrease in adiponectin (adipose tissue and spleen) and an increase in TNF-alpha (spleen), IL-6 (spleen), and PAI-1 (adipose tissue, spleen) in the OB animals were observed, showing an inflammatory status in this group. These changes may negatively influence glucose homeostasis and lipid profile, AP, clotting, and fibrinolysis, which may lead to endothelial dysfunction, and atherosclerosis (Tapsell et al., 2004). Adiponectin exerts beneficial effects in type 2 diabetic patients since it is related to increased insulin sensitivity in a variety of tissues (Tapsell et al., 2004). In contrast, IL-6 and PAI-1 are directly related to diabetes mellitus. IL-6 for example, stimulates the inhibition of insulin action, leading to increased glucose (Hotamisligil et al., 1994; Fernandez-Real and Ricart, 2003). Moreover, IL-6 interferes with the anti-inflammatory effect of insulin and may also cause inhibition of the insulin signal transduction (Hotamisligil et al., 1994; Kajitani et al., 2010). The action of IL-6 may be considered a “two-way street,” since high levels of IL-6 may be regarded as an insulin resistance predictor of endothelial function and hence may be involved in the development of type 2 diabetes (Juhan-Vague et al., 1991).

In turn, the PAI-1 has been associated with increased insulin concentrations and decreased insulin sensitivity (Juhan-Vague et al., 1991). Furthermore, Juhan-Vague et al. (1991) suggested that increased PAI-1 concentrations may be the connection between insulin resistance and coronary disease. Thus, our findings pointing to increased glucose, triglycerides, and glucose intolerance observed in the OB group may be related to decreased adiponectin and increased IL-6, TNF-alpha, and PAI-1. Importantly, we observed negative correlations between baroreflex sensitivity and spleen PAI-1 and IL-6 levels and between RMSSD and PAI-1 in spleen tissue. Moreover, spontaneous baroreflex and RMSSD were positively correlated with adiponectin.

In addition to an increased inflammatory profile in the obese animals, we observed, in the heart and kidneys, a decrease in antioxidant capacity and an increase in membrane lesion, as demonstrated by increased lipoperoxidation (TBARS) and protein oxidation (carbonyls). The induced oxidative damage in cells and tissues has been linked to the etiology of various degenerative diseases such as heart disease, atherosclerosis, and pulmonary disorders (Ames et al., 1993). It is also worth mentioning that there was a decrease in CAT in OB animals, suggesting an impairment of redox balance in obese animals. Additionally, in the present study, we observed that the higher levels of inflammatory mediators (PAI-1 and TNF-alpha in spleen tissue) were correlated with higher levels of protein oxidation (cardiac tissue) and lipoperoxidation (renal and cardiac tissues).

Beside the negative effects of hyperglycemia on estrogen activity and expression, which could other possible explanation.

Our study has limitations that deserve comments. We have methodological limitations regarding cardiac function measurements, since that we not confirmed echocardiographic parameters by direct left ventricular catheterization, tissue Doppler, or magnetic resonance imaging. Although we observed important relationships between metabolic, inflammatory, and autonomic parameters, a cause-effect relationship cannot be established. Regarding the model, the ob/ob mice present a nonsense mutation (C to T) in codon 105 changes an Arg residue to a stop codon causing premature truncation, rendering the translated protein biologically inactive. Although there are high levels of leptin mRNA in adipocytes, the animals completely lack functional leptin (Friedman et al., 1991; Zhang et al., 1994). Despite the ob/ob show similar type 2 diabetes complication as seen human, as obesity, hyperglycemia, insulin resistance, diabetic neuropathy, as well as decreased baroreflex sensitivity, the etiology of these complications are different in human and ob/ob mice. It is important to emphasize that we used young animals instead aged animals, which could mimic more the human situation. However, our intention was to show the effects of the obesity and diabetes in females, avoiding the possible effects of the aging. It is worth to consider that in the last two decades, type 2 diabetes, once thought to be a metabolic disorder exclusively of adulthood, has become increasingly more frequent in obese adolescents (Pinhas-Hamiel and Zeitler, 2005).

## Conclusions

In conclusion, our results show that adult female ob/ob mice presented diastolic dysfunction associated with cardiovascular autonomic impairment, which may have triggered inflammation and oxidative stress. These findings may shed some light on the mechanisms involved in the diastolic dysfunction development promoted by the association of diabetes and obesity in the female gender, which has conjectured a higher risk of developing cardiovascular complication in females when compared to males. It is likely a consequence of a greater impairment on endothelial response due to diabetes in females, which promotes an alteration on insulin and estrogen signaling (reducing the benefits of the estrogen on the cardiovascular system), leading to an increased oxidative stress and inflammation as observed in this study. Additionally, anti-obesity and anti-inflammatory therapeutic interventions, as weight loss and exercise training as well as new approaches considering the physiological particularities of the female sex should be considered in the management of the metabolic disorders also in adult female subjects in which the protective action of hormones are still present.

## Author contributions

MS: Contributed to conception and design of the work, acquisition of data, analysis and interpretation of data, statistical analysis, and draft the manuscript; FC: Contributed to interpretation of data, statistical analysis, and draft the manuscript; DD: Contributed to acquisition of data, analysis and interpretation of data; FS: Contributed to acquisition of data, analysis and interpretation of data; JM: Contributed to acquisition of data, analysis and interpretation of data; ZP: Contributed to acquisition of data, analysis and interpretation of data; DC: Contributed to acquisition of data, analysis and interpretation of data; BR: Contributed to interpretation of data, and draft the manuscript; KD: Contributed to conception and design of the work, analysis and interpretation of data, statistical analysis and draft the manuscript; MI: Contributed to conception and design of the work, interpretation of data, and draft the manuscript.

### Conflict of interest statement

The authors declare that the research was conducted in the absence of any commercial or financial relationships that could be construed as a potential conflict of interest.
